# User-Side Long-Baseline Undifferenced Network RTK Positioning Under Geomagnetic Storm Conditions Using a Power Spectral Density-Constrained Ionospheric Delay Model

**DOI:** 10.3390/s25206433

**Published:** 2025-10-17

**Authors:** Yixi Wang, Huizhong Zhu, Qi Xu, Jun Li, Chuanfeng Song, Bo Li

**Affiliations:** 1 School of Geomatics, Liaoning Technical University, Fuxin 123000, China; 2 College of Surveying and Mapping Engineering, Heilongjiang Institute of Technology, Harbin 150050, China

**Keywords:** URTK, geomagnetic storm, long baseline, ambiguity resolution, atmospheric parameter estimation, ionospheric delay

## Abstract

To address the problem of the degraded positioning accuracy of the long-baseline undifferenced network RTK (URTK) under extreme space weather conditions, herein, we propose a user-side atmospheric delay estimation strategy based on the undifferenced network RTK concept to enhance positioning performance in geomagnetic storm environments. First, an ambiguity-resolution model that jointly estimates atmospheric error parameters is used to fix the carrier-phase integer ambiguities for long-baseline reference stations. The accurately fixed inter-station ambiguities are then linearly transformed to recover station-specific undifferenced integer ambiguities; undifferenced observation errors at each reference station are computed to generate corresponding undifferenced correction terms. Lastly, recognizing that ionospheric delays vary sharply during geomagnetic storms and can severely compromise the availability of regional undifferenced correction models, we estimate the residual atmospheric parameters on the user side after applying regional corrections. Experimental results show that the server side is not significantly impacted during geomagnetic storms and can continue operating normally. On the user side, augmenting the solution with atmospheric parameter estimation effectively improves positioning availability. Under strong geomagnetic storms, the proposed mode improves user-station positioning accuracy by 63.7%, 60.7%, and 64.4% in the east (E), north (N), and up (U) components, respectively, relative to the conventional user-side solution; under moderate storm conditions, the corresponding improvements are 16.7%, 10.0%, and 11.1%.

## 1. Introduction

Real-time kinematics (RTK) is a key technique in achieving dynamic, high-accuracy positioning. However, as the user–reference baseline length increases, the spatial correlation of atmospheric delays weakens; thus, these errors can no longer be eliminated via differencing. Consequently, the operational coverage of conventional RTK typically does not exceed ~30 km; in comparison, network RTK (NRTK) can effectively extend the service area [1]. As the most widely deployed regional augmentation technique, NRTK generally comprises three modules, namely reference station ambiguity fixing, regional error modeling and correction, and user-side positioning [2]. Nevertheless, NRTK still faces practical limitations: dense reference networks are required in atmospherically active regions; station deployment is difficult in remote areas; and users often need to provide a coarse a priori position to the server. Thus, enlarging the service coverage while ensuring reliable, high-accuracy positioning for users remains an ongoing issue in employing NRTK.

Compared with the leading precise point positioning–real-time kinematics (PPP-RTK) regional augmentation framework, NRTK can achieve centimeter-level positioning over large areas without relying on external differential code bias (DCB) and uncalibrated phase delay (UPD) products and therefore remains one of the most practical augmentation solutions. On the user side, two modeling strategies are commonly adopted, which are double-differenced and undifferenced. The double-differenced approach is algorithmically simple. Double-differenced ambiguities are easier to fix and converge faster; however, the resulting measurement noise level is roughly twice that of the undifferenced model. The undifferenced approach supports longer operating distances and yields higher ultimate accuracy, albeit with greater implementation complexity [3,4,5,6,7].

In recent years, undifferenced and long-baseline network RTK has become a focal topic in high-precision GNSS positioning. The authors of Ref. [8] first systematically proposed an undifferenced network RTK (URTK) method in 2013, which achieves centimeter-level positioning accuracy through undifferenced error correction and the rapid resolution of single-differenced ambiguities. The authors of Ref. [9] developed a long-baseline URTK algorithm using BeiDou Navigation Satellite System (BDS) data, combining multi-frequency integer ambiguity resolution with undifferenced error corrections to realize high-accuracy positioning; their results indicate that BDS-3 outperforms global positioning system (GPS) in both positioning accuracy and convergence time, achieving centimeter-level solutions. The authors of Ref. [10] present an undifferenced, uncombined ambiguity resolution approach for long baselines between BDS reference stations that exploits multi-frequency observations, zenith delay estimation, and real-time linear computation, effectively improving the success rate of integer fixing; validations demonstrated faster and more accurate solutions than conventional methods. The authors of Ref. [11] introduced an undifferenced zenith-tropospheric-delay (ZTD) prediction model for mitigating tropospheric errors in continuously operating reference station (CORS) networks; experiments showed reduced tropospheric error, improved float ambiguity precision, a 45% reduction in recovery time, and significantly enhanced ambiguity restoration over long baselines. The authors of Ref. [12] analyzed how variations in geomagnetic and ionospheric conditions degrade the accuracy of network ionospheric corrections for long-baseline RTK; under strong ionospheric storms, correction accuracy decreases, instantaneous ambiguity resolution (AR) fails, and kinematic AR requires more time to re-fix integers.

Under extreme space weather conditions—especially during geomagnetic storms—GNSS signals traversing the ionosphere experience sharp variations in total electron content (TEC), leading to increased observation noise, frequent cycle slips, and even signal outages, thereby degrading convergence and positioning accuracy for high-precision GNSSs [13,14]. From a physical perspective, geomagnetic storms induce energetic particle precipitation and Joule heating, leading to phenomena such as sharp increases in TEC, enhanced ionospheric irregularities, and complex spatiotemporal variations even at mid- and low latitudes. These physical processes are the fundamental causes of the failure of network RTK regional correction models. The authors of Ref. [15] compared ionospheric prediction models for GPSs, Galileo, and BDS against International GNSS Service (IGS) post-processed products; during strong storms, forecast performance deteriorates, and the Klobuchar–GPS model is recommended when Galileo/BDS signals are unavailable. The authors of Ref. [16] examined the impacts of the extreme geomagnetic storm in May 2024 on the ionosphere and GPS kinematic precise point positioning (PPP). The auroral oval underwent pronounced equatorward expansion, reaching unusually low geomagnetic latitudes, rate of TEC index (ROTI) peaks reached four times the background level, and positioning errors increased by factors of 1.5–5 near the auroral oval boundary, significantly affecting navigation and communications. The authors of Ref. [17] analyzed the lagged ionospheric response to two strong geomagnetic storms and its impact on kinematic PPP, highlighting the complex coupling among the solar wind, magnetosphere, and ionosphere. The authors of Ref. [18] investigated the impact of ionospheric disturbances on GNSS positioning during geomagnetic storms at high latitudes. The authors found that positioning errors increase exponentially with the rise in the TEC index.

However, the authors of existing studies have mainly focused on post-event analyses or PPP performance enhancement using ionospheric products such as TEC and ROTI. For network RTK, which inherently operates in real-time, previous studies have mainly focused on post-event analyses or PPP performance enhancement. However, few have systematically investigated how to improve user-side algorithms to maintain stable centimeter-level positioning accuracy under extreme geomagnetic storm conditions in low-latitude regions. In this study, we use data from a low-latitude provincial CORS network in China on Day of Year (DOY) 309, 2023 (5 November 2023) and DOY 226, 2024 (13 August 2024) to (i) fix carrier-phase integer ambiguities for the reference network using an atmospheric constraint model and convert them to undifferenced integer ambiguities; (ii) compute undifferenced error corrections at each reference station, interpolate them, and broadcast them to users; and (iii) improve user-side ambiguity resolution during storms by augmenting the model with additional atmospheric parameter estimation, thereby mitigating residual atmospheric delays that persist after applying regional undifferenced corrections and that otherwise hinder correct integer fixing.

Previous studies by Li et al. (2023, GPS Solutions) and Li et al. (2023, Remote Sensing) established the theoretical foundation for constraining atmospheric parameters using the power spectral density (PSD) characteristics of ionospheric and tropospheric variations [19,20]. Those studies primarily focused on modeling and validation at the network or server side, demonstrating the feasibility of PSD-based stochastic constraint models in improving long-baseline ambiguity resolution. Building upon this foundation, the present study aims to evaluate a long-baseline undifferenced network RTK (URTK) approach incorporating user-side atmospheric parameter estimation under both strong and moderate geomagnetic storm conditions. The results provide practical insights for achieving high-precision real-time positioning services under extreme space weather environments.

## 2. Long-Baseline Undifferenced NRTK: Mathematical Model

### 2.1. Undifferenced Observation Equations

GNSS observations describe the spatial geometric range between the satellite receiver geometric range, and each observation exists independently [21]. During signal propagation, the signal is affected by various errors; therefore, the observation equations can intuitively express the relationships between them. The undifferenced observation equations for the linearized pseudorange and carrier-phase measurements can be expressed as follows [22]:

(1) $$L_{r,j}^{s}=\rho_{r}^{s}+T_{r}^{s}+dt_{r}+\lambda_{j}\delta_{r,j}-dt^{s}-\lambda_{j}\delta_{j}^{s}-\mu_{j}\cdot I_{r}^{s}+\lambda_{j}N_{r,j}^{s}+\varepsilon_{L_{r,j}^{s}}$$

(2) $$P_{r,j}^{s}=\rho_{r}^{s}+T_{r}^{s}+dt_{r}+d_{r,j}-dt^{s}-d_{j}^{s}+\mu_{j}\cdot I_{r}^{s}+\varepsilon_{P_{r,j}^{s}}$$

where $L_{r,j}^{s}$ and $P_{r,j}^{s}$ denote the undifferenced carrier-phase and pseudorange observables (in meters); $\rho_{r}^{s}$ is the geometric range from the station to the satellite; r, s, and j denote the station, the satellite, and the frequency; $T_{r}^{s}$ is the tropospheric delay parameter along the propagation path; $I_{r}^{s}$ is the ionospheric delay at the first frequency along the propagation path, with $\mu_{j}=f_{1}^{2}/f_{j}^{2}$; $f_{j}$ denotes the j-th frequency; $\lambda_{j}=c/f_{j}$ is the carrier-phase wavelength corresponding to frequency j; and $dt_{r}$ and $dt^{s}$ are the receiver and satellite clock offsets, respectively. For the phase data, also present are the receiver phase bias $\delta_{r,j}$ and the satellite phase bias $\delta_{j}^{s}$, both expressed in cycles; in the corresponding pseudorange data, the receiver code (pseudorange) bias $d_{r,j}$ and the satellite code bias $d_{j}^{s}$ are present, both expressed in meters; $d_{r,j}$ is the integer ambiguity expressed in cycles; and $\varepsilon_{L_{r}^{s}}$ and $\varepsilon_{P_{r}^{s}}$ are the measurement noises of the pseudorange and carrier-phase observations, respectively. Building on the undifferenced observation equations, applying the between-satellite single difference and the between-station (between-receiver) single difference yields the receiver satellite double-difference model.

In this paper, atmospheric delay errors are treated as a random walk, with the epoch-to-epoch means of both ionospheric and tropospheric differences set to zero; by tuning the variances associated with these means, the atmospheric parameters can be constrained. Before estimating the tropospheric delay error via a mapping (projection) function, an a priori tropospheric model can be applied for correction. The random walk process for the tropospheric delay parameter can be expressed as

(3) $$RZTD(t)-RZTD(t-1)=\omega_{Z}(t),\omega_{Z}(t)\;\sim \;N(0,\sigma_{Z}^{2})$$

In the equation, RZTD(t) denotes the ZTD at epoch t; t−1 denotes that at the previous epoch; and $\omega_{Z}(t)$ denotes the inter-epoch increment of the zenith tropospheric parameter, which is modeled as a zero-mean Gaussian with variance $\sigma_{Z}^{2}$. The troposphere varies relatively smoothly, and the power spectral density of the random walk process is generally set to an empirical value [23].

The temporal variation in the ionospheric delay exhibits random walk characteristics and can be described by a random walk:

(4) $$I_{r,1}^{s}(t)-I_{r,1}^{s}(t-1)=\omega_{I},\omega_{I}\;\sim \;N(0,\sigma_{I}^{2})$$

$I_{r,1}^{s}(t)$ denotes the inter-station single-difference ionospheric delay at epoch t and $\omega_{I}$ denotes the ionospheric parameter, modeled as a zero-mean Gaussian with variance $\sigma_{I}^{2}$. From Equations (3) and (4), it follows that determining an appropriate variance for the atmospheric delay is sufficient to impose suitable constraints on the atmospheric parameters.

The ionospheric variance depends on the time interval and the power spectral density; the relationship between the variance and the power spectral density is as follows:

(5) $$\sigma_{I}^{2}=\Delta t\cdot q^{2}(I_{r,1}^{s})$$

The effectiveness of constraining the time-varying characteristics of the ionospheric parameters hinges on the choice of the power spectral density (PSD). The method for obtaining a PSD that reflects the ionosphere’s temporal variability has been described in detail in [19,20] and is not repeated herein. Below, we present the specific pseudo-observation equation for the random walk process of the atmospheric parameters, coupled with the correspondence among the PSD, the variance, and the weight of the pseudo-observation equation, as follows:

(6) $$\left\{\begin{array}{c} v_{Z}=RZTD(t)-RZTD(t+1),\gamma_{Z}=\frac{\sigma_{0}^{2}}{\sigma_{Z}^{2}}=\frac{\sigma_{0}^{2}}{q_{Z}^{2}\cdot \Delta t}\\ v_{I}=I_{r,1}^{s}(t)-I_{r,1}^{s}(t+1),\gamma_{I}=\frac{\sigma_{0}^{2}}{\sigma_{I}^{2}}=\frac{\sigma_{0}^{2}}{q^{2}(I_{r,1}^{s})\cdot \Delta t} \end{array}\right.$$

In the equation, $v_{Z}$ and $v_{I}$ denote, respectively, the zenith tropospheric and ionospheric residuals; $\gamma_{Z}$ and $\gamma_{I}$ denote the weights of the pseudo-observation equations separately for the tropospheric and ionospheric delays; $q_{Z}^{2}$ denotes the tropospheric power spectral density; $\sigma_{0}^{2}$ denotes the unit weight standard deviation; and Δt denotes the time interval of atmospheric variation. It should be noted that Equations (3) and (4) describe the stochastic evolution of atmospheric parameters in the form of a random walk process between consecutive epochs, whereas Equation (6) represents the implementation of these temporal constraints within the least-squares estimation framework. Specifically, Equation (6) transforms the random walk process model into a pseudo-observation equation so that the temporal correlation between adjacent epochs can be effectively utilized as additional constraints. In other words, Equations (3) and (4) define the process model, and Equation (6) expresses that model in observation form for parameter estimation. Accordingly, the ambiguity observation model that accounts for the random walk of atmospheric delays can be represented as follows:

(7) $$\left\{\begin{array}{c} L_{r,1}^{s,1}=Map_{r}^{s,1}RZTD_{r,1}+\lambda_{j}(N_{r,1}^{s}-N_{r,1}^{1})-MF_{r,1}^{s}I^{s}+MF_{r,1}^{1}I^{1}\\ l_{r,1}^{s,1}=Map_{r}^{s,1}RZTD_{r,1}+MF_{r,1}^{s}I^{s}-MF_{r,1}^{1}I^{1} \end{array}\right.$$

where L denotes the constant term, Map the tropospheric mapping function, and MF the ionospheric mapping function. From Equation (7), it can be seen that, compared with the conventional double-difference ambiguity resolution model, the ionospheric delay and ambiguity parameters are transformed from the double-difference form to the between-satellite single-difference form. From Equations (6) and (7), the ambiguity resolution model with an inter-epoch random walk constraint on the additional atmospheric parameters can be derived as follows:

(8) $$\left[\begin{array}{ccccc} E_{sys}⊗Map & 0 & -E_{sys\times sys}⊗MF & 0 & E_{sys\times sys}⊗\lambda \\ E_{sys}⊗Map & 0 & E_{sys\times sys}⊗MF & 0 & 0\\ 1 & -1 & 0 & 0 & 0\\ 0 & 0 & E_{sat\times sat} & -E_{sat\times sat} & 0 \end{array}\right]\left[\begin{array}{c} RZTD_{t}\\ RZTD_{t+1}\\ I_{t}\\ I_{t+1}\\ N \end{array}\right]-\left[L\right]=\left[V\right]$$

where

$$\begin{array}{l} MF=\left[\begin{array}{ccccc} -MF_{r,1}^{1} & MF_{r,1}^{2} & 0 & \ldots & 0\\ -MF_{r,1}^{1} & 0 & MF_{r,1}^{3} & \ldots & 0\\ \vdots & \vdots & \vdots & \ddots & 0\\ -MF_{r,1}^{1} & 0 & 0 & \ldots & MF_{r,1}^{j}\\ \vdots & \vdots & \vdots & \vdots & \vdots \\ -\frac{f_{1}^{2}}{f_{i}^{2}}MF_{r,1}^{1} & 0 & 0 & \ldots & \frac{f_{1}^{2}}{f_{i}^{2}}MF_{r,1}^{j} \end{array}\right]\\ \lambda =\left[\begin{array}{ccc} \lambda_{1}b & \ldots & 0\\ \vdots & \ddots & \vdots \\ 0 & \ldots & \lambda_{i}b \end{array}\right]\;b=\left[\begin{array}{ccccc} -1 & 1 & 0 & \ldots & 0\\ -1 & 0 & 1 & \ldots & 0\\ \vdots & \vdots & \vdots & \ddots & \vdots \\ -1 & 0 & 0 & \ldots & 1 \end{array}\right] \end{array}$$

In the equation, $E_{num}$ is an all-ones matrix conformable with num×1; $E_{num\times num}$ is the identity matrix conformable with num×num; ⊗ denotes the Kronecker product; sys is the number of satellite navigation systems (constellations); sat is the total number of observed satellites for the corresponding system; and V is the residual of each observation equation. Using the relation $I_{j}=\left(f_{1}^{2}/f_{j}^{2}\right)I_{1}$, the number of ionospheric parameters is reduced; therefore, only the ionospheric parameter at the first frequency must be estimated. In addition, inter-epoch random walk constraints on the atmospheric parameters are incorporated into the observation equations to accelerate the convergence of the ambiguity parameters. The observation equation for the pseudorange is analogous to that for the carrier phase, except that it lacks the integer ambiguity parameter. Because the inter-station single-difference ionospheric parameter and the inter-station single-difference ambiguity parameter of the reference satellite are strongly correlated with the corresponding parameters of the other satellites, a datum must be imposed to avoid rank deficiency; in this case, these two parameters of the reference satellite are set to zero. Least-squares estimation is used to obtain the float solutions and variance–covariance matrix of the position, atmospheric delay, and ambiguity parameters. Subsequently, the integer ambiguities are searched and fixed using the LAMBDA (Least-squares AMBiguity Decorrelation Adjustment) method [24]. The correctness of ambiguity fixing is assessed using the ratio test, with an empirical threshold set to ratio >2.0. For satellites that fail the fixing process, the float solutions are retained and flagged, excluding them from the subsequent generation of correction terms to ensure the reliability of the broadcast corrections [25].

### 2.2. Method for Generating Undifferenced Corrections

First, after transforming the double-differenced integer ambiguities at the reference stations into undifferenced integer ambiguities and fixing them, the undifferenced error corrections for each station can be computed. Assuming three reference stations A, B, and C, the undifferenced error correction (observed-minus-computed, OMC) for satellite P can be expressed as follows:

(9) $$\left\{\begin{array}{c} OMC_{A,i}^{p}=\lambda_{i}\varphi_{A,i}^{p}-\rho_{A}^{p}=c\left(t_{A}-t^{p}\right)+\lambda_{i}N_{A,i}^{p}+(\delta_{A,i}-\delta_{i}^{p})-I_{A,i}^{p}+T_{A}^{p}+\varepsilon_{A,i}^{p}\\ OMC_{B,i}^{p}=\lambda_{i}\varphi_{B,i}^{p}-\rho_{B}^{p}=c\left(t_{B}-t^{p}\right)+\lambda_{i}N_{B,i}^{p}+(\delta_{B,i}-\delta_{i}^{p})-I_{B,i}^{p}+T_{B}^{p}+\varepsilon_{B,i}^{p}\\ OMC_{C,i}^{p}=\lambda_{i}\varphi_{C,i}^{p}-\rho_{C}^{p}=c\left(t_{C}-t^{p}\right)+\lambda_{i}N_{C,i}^{p}+(\delta_{C,i}-\delta_{i}^{p})-I_{C,i}^{p}+T_{C}^{p}+\varepsilon_{C,i}^{p} \end{array}\right.$$

Note that the correction term adjusts the satellite receiver geometric LOS range and incorporates modellable effects (e.g., hydrostatic troposphere, Sagnac correction, multipath). Next, the observation corrections at each station can be adjusted for the undifferenced integer ambiguities to obtain the undifferenced error correction Cor:

(10) $$\left\{\begin{array}{c} Cor_{A,i}^{p}=OMC_{A,i}^{p}-\lambda_{i}N_{A,i}^{p}=c\left(t_{A}-t^{p}\right)+(\delta_{A,i}-\delta_{i}^{p})-I_{A,i}^{p}+T_{A}^{p}+\varepsilon_{A,i}^{p}\\ Cor_{B,i}^{p}=OMC_{B,i}^{p}-\lambda_{i}N_{B,i}^{p}=c\left(t_{B}-t^{p}\right)+(\delta_{B,i}-\delta_{i}^{p})-I_{B,i}^{p}+T_{B}^{p}+\varepsilon_{B,i}^{p}\\ Cor_{C,i}^{p}=OMC_{C,i}^{p}-\lambda_{i}N_{C,i}^{p}=c\left(t_{C}-t^{p}\right)+(\delta_{C,i}-\delta_{i}^{p})-I_{C,i}^{p}+T_{C}^{p}+\varepsilon_{C,i}^{p} \end{array}\right.$$

From Equation (10), the undifferenced error correction primarily contains dispersive errors dominated by ionospheric delay and non-dispersive errors dominated by tropospheric delay (in addition to satellite and receiver clock offsets, satellite and receiver hardware delays, etc.). After obtaining the undifferenced error corrections independently for each reference station, the user’s undifferenced corrections can be computed through interpolation modeling. When the spacing of the reference network is large, separate error models may be established for the ionospheric delay and the neutral atmosphere delay, and the corresponding errors are interpolated to the user’s location [26].

### 2.3. User-Side Treatment of Residual Atmospheric Errors During Geomagnetic Storms

In conventional NRTK algorithms, the user-side processing assumes that the regional undifferenced correction model has already corrected errors such as ionospheric delay; thus, the user solution typically does not further handle ionospheric parameters. As shown in the previous subsection, however, ionospheric delays vary sharply during geomagnetic storms, increasing the number of errors produced by the regional undifferenced correction model and potentially leaving measurements only partially corrected [12,27,28,29,30]. Therefore, by extending the ambiguity resolution strategy used on the server side—where atmospheric parameters are explicitly estimated and constrained through a random walk model—to the user side, residual atmospheric parameters can be further estimated to decouple atmospheric errors from the integer ambiguity parameters and enhance user-side performance. Based on the model formulation presented in Section 2.1, the corresponding user-side ambiguity resolution model with atmospheric parameter estimation is expressed as follows:

(11) $$\left[\begin{array}{cccc} A_{sys} & E_{sys}⊗Map & -E_{sys\times sys}⊗MF & E_{sys\times sys}⊗\lambda \\ A_{sys} & E_{sys}⊗Map & E_{sys\times sys}⊗MF & 0 \end{array}\right]\left[\begin{array}{c} X\\ RZTD\\ I\\ N \end{array}\right]-[L]=[V]$$

The difference between Equations (8) and (11) is that the set of unknowns in Equation (11) additionally includes the three position parameters, the relative zenith tropospheric parameter, and the ionospheric delay parameter; moreover, no inter-epoch random walk constraint is imposed on the atmospheric parameters in this equation.

## 3. Case Studies and Analysis

To comprehensively evaluate the impact of major geomagnetic storms on the positioning performance of long-baseline network RTK, we selected the geomagnetic storm events that occurred on 5 November 2023 (DOY 309) and 13 August 2024 (DOY 226) as the research objects. Internationally, geomagnetic storm intensity is usually measured using the geomagnetic activity indices Dst, Kp, and ap [31]. The Dst index reflects the strength of global magnetic field disturbances and is an important indicator for assessing storm intensity; the Kp and ap indices mainly describe the global average level of geomagnetic disturbances, with ap being a linear transformation of Kp. However, Kp and ap are generally updated every 3 h and thus have low temporal resolution, making it difficult to capture rapidly changing geomagnetic conditions. Therefore, to describe geomagnetic activity in finer detail, we introduce the hourly ap60 and Hp60 indices. Of the two, ap60 provides a higher-temporal-resolution representation of global geomagnetic disturbances; in comparison, Hp60 mainly reflects the magnitude of magnetic field variations.

**Figure 1:**
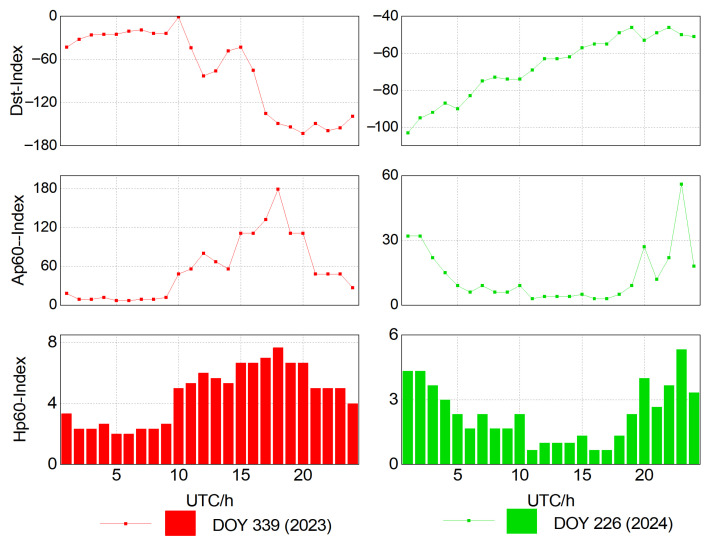
Geomagnetic storm activity indices on DOY 309 (2023) and DOY 226 (2024).

We collected Dst, ap60, and Hp60 data for the strong geomagnetic storm period in 2023 (DOY 309) and the moderate geomagnetic storm period in 2024 (DOY 226) (data sources: https://wdc.kugi.kyoto-u.ac.jp/ and https://kp.gfz.de/). The results presented in Figure 1 show the variations in geomagnetic activity indices over the two days. As can be seen, during DOY 309 in 2023, the minimum Dst dropped to −163 nT, the peak ap60 reached 179, and the peak Hp60 reached 7.667. During the moderate storm on DOY 226 in 2024, the minimum Dst dropped to −103 nT, the peak ap60 reached 56, and the peak Hp60 reached 5.333.

**Figure 2:**
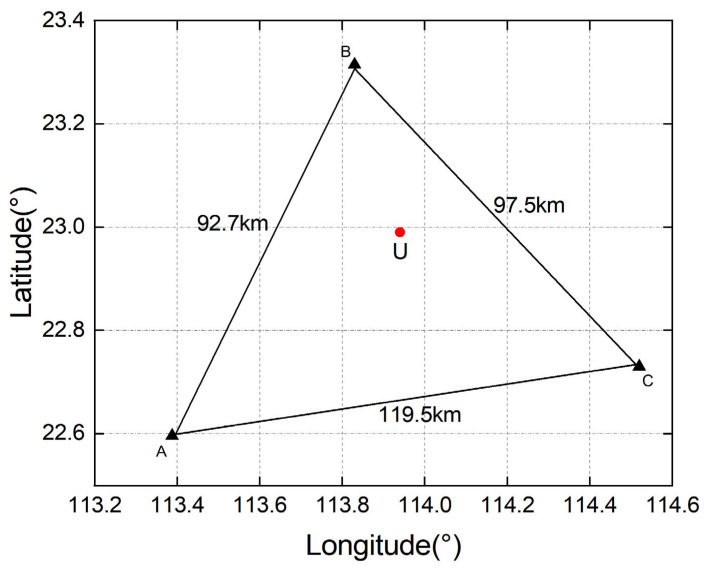
Layout of reference stations and user station.

We first apply a self-developed long-baseline URTK algorithm to perform dual-constellation (BDS + GPS), multi-frequency URTK processing on long-baseline observations collected under geomagnetic storm conditions. The experiments use CORS network data from a low-latitude province in southern China on DOY 309 (2023) and DOY 226 (2024), with a sampling interval of 1 s, a daily observation span of 24 h, and an elevation mask angle of 15°, and the carrier-to-noise ratio threshold was set to 45 dB-Hz. Cycle slips were detected using the Melbourne–Wübbena (M–W) method, and the data were processed in simulated kinematic positioning mode. The observing network consists of three reference stations, A, B, and C, and a user station, U. The inter-station distances are listed in Table 1: baseline AB is 92.7 km, BC is 97.5 km, and AC is 119.5 km; the diagram presented in Figure 2 displays the layout of the reference stations and the user station.

**Figure 3:**
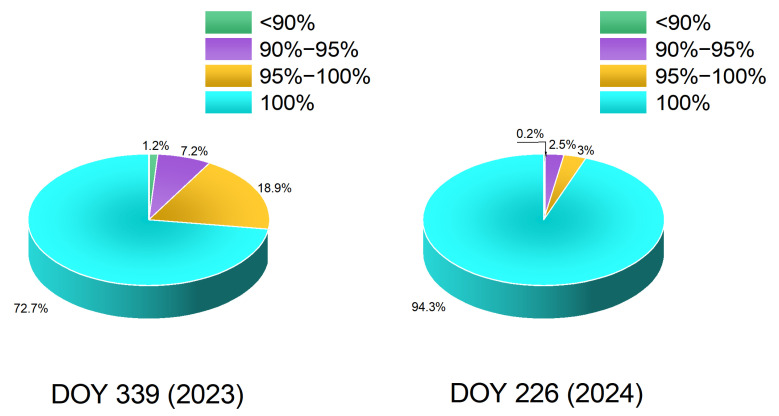
Inter-station ambiguity fix rate statistics on DOY 309 (2023) and DOY 226 (2024).

Accurate computation of the reference network carrier-phase integer ambiguities is the core issue for network RTK algorithms under geomagnetic storm conditions, and it is also key in this paper to the conversion from double-difference ambiguities to undifferenced ambiguities for generating undifferenced error corrections. Because the server-side integer ambiguities provide essential support for the subsequent regional undifferenced correction model and for high-precision user positioning, we first examine whether geomagnetic storms affect the server side. To quantify the fix rate, the proportions of ambiguity fix rates across different intervals are given in Figure 3. The fix rate is defined as the ratio, at the current user epoch, of the number of satellites that pass the integer-ambiguity closure test to the total number of satellites participating in integer fixing at that epoch. As shown in Figure 3, due to the relatively intense geomagnetic activity on DOY 309 (2023), the reference station ambiguity fix rate is lower than on DOY 226 (2024). Nevertheless, for both days, most of the reference station ambiguity fix rates exceed 90%. To further assess the reliability of ambiguity fixing, a “correctly ambiguity fix rate” was defined and evaluated for each inter-station baseline. This metric quantifies the proportion of correctly fixed integer ambiguities among all epochs where ambiguity fixing was attempted. Specifically, an ambiguity fixing result is considered correct if its post-fit residuals satisfy the closure test and remain within 1.5 times the observation noise level, indicating consistent integer resolution. The correct fix rate is then calculated as

(12) $$CF=\frac{N_{c}}{N_{f}}\times 100\%$$

where CF denotes the correctly fixed ambiguity rate, $N_{c}$ denotes the number of epochs with correctly fixed ambiguities, and $N_{f}$ denotes the total number of epochs with fixed ambiguities.

**Figure 4:**
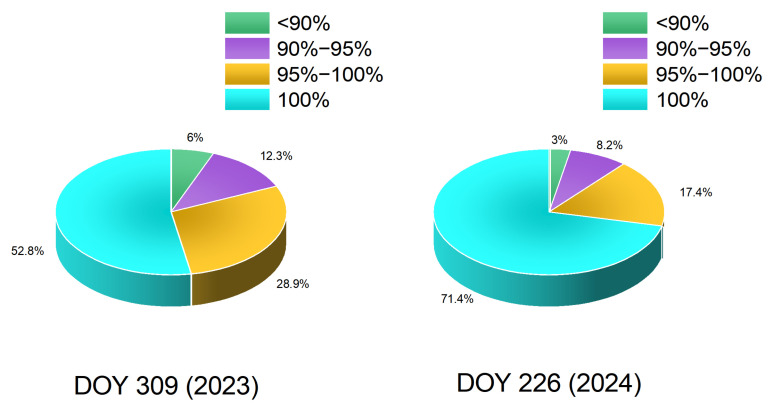
Inter-station correct ambiguity fix rate statistics on DOY 309 (2023) and DOY 226 (2024).

The correctly fixed ambiguity rates for DOY 309 (2023) and DOY 226 (2024) are shown in Figure 4. Similarly to Figure 3, the legend represents the specific proportions of correctly fixed ambiguity rates in different intervals. As illustrated, some ambiguities were incorrectly fixed due to the occurrence of geomagnetic storm activity, indicating that geomagnetic storms exert a certain impact on ambiguity fixing between reference stations.

**Figure 5:**
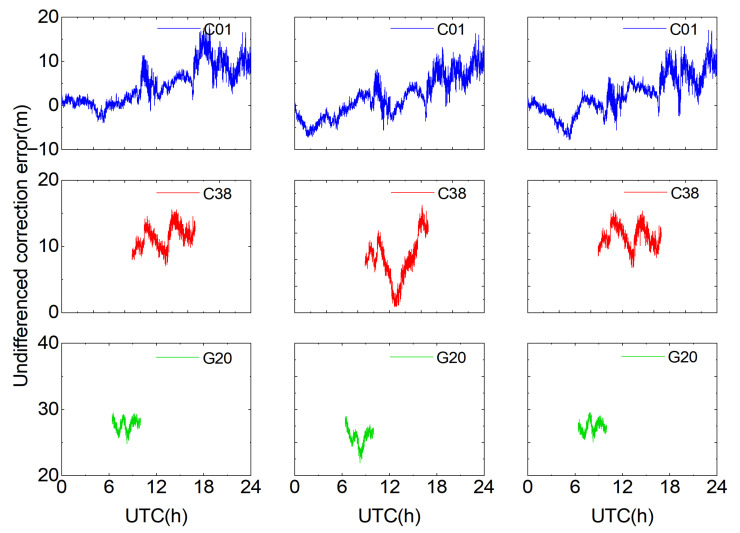
Undifferenced error corrections for selected satellites on DOY 309 (2023).

**Figure 6:**
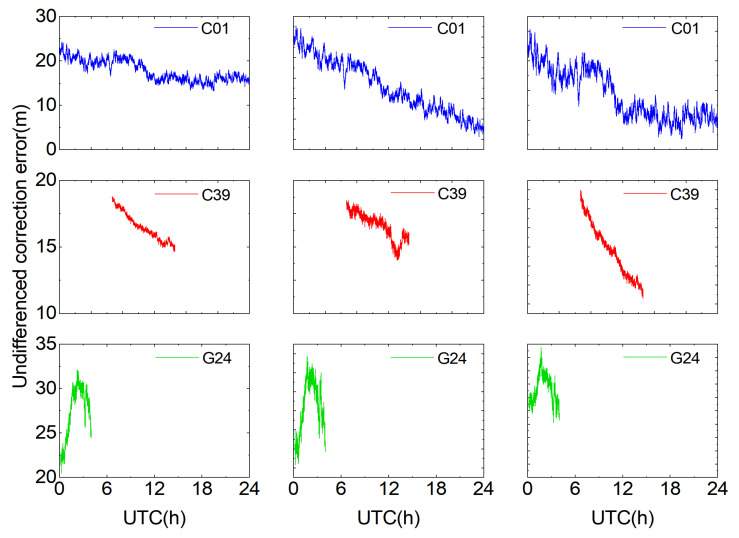
Undifferenced error corrections for selected satellites on DOY 226 (2024).

To compute the user’s undifferenced error corrections, the accurately determined double-difference integer ambiguities are first converted to undifferenced integer ambiguities, after which the undifferenced composite error corrections for each reference station are obtained. Taking as examples the periods of continuous visibility on the first frequency for satellites C01, C38, and G20 on DOY 309 (2023) and satellites C01, C39, and G24 on DOY 226 (2024) at reference stations A, B, and C (Figure 5 and Figure 6), it can be seen that each satellite’s undifferenced corrections vary approximately linearly, and the trends for the same satellite are similar across the three stations. Because all reference stations lie within a small region, the atmospheric variations are similar, resulting in similar undifferenced error corrections at each station. For satellite C01 on DOY 309 (2023), satellite C01 on DOY 226 (2024), and satellite G24 on DOY 226 (2024), the undifferenced error corrections vary more sharply during periods when the Dst index is high and geomagnetic storms occur.

For long-baseline network RTK positioning, the highly variable ionospheric conditions induced by geomagnetic storms can significantly degrade positioning accuracy. Consequently, the interpolation of ionospheric delay errors becomes a key factor affecting the effectiveness of user correction. To better understand the ionospheric delay variation contained in the user-side undifferenced correction terms, a classified interpolation method is employed to extract the double-differenced ionospheric delay error component from the undifferenced corrections.

In network RTK, undifferenced correction terms can be directly applied for user error correction; however, in long-baseline scenarios, such corrections may be inaccurate. Therefore, it is necessary to classify the undifferenced correction information according to error characteristics before interpolation. The classified interpolation method performs the interpolation of undifferenced non-dispersive errors on the Earth’s surface, where the reference and user stations are located, whereas the interpolation of undifferenced ionospheric delay errors for the rover station is conducted on a plane corresponding to the effective ionospheric height.

**Figure 7:**
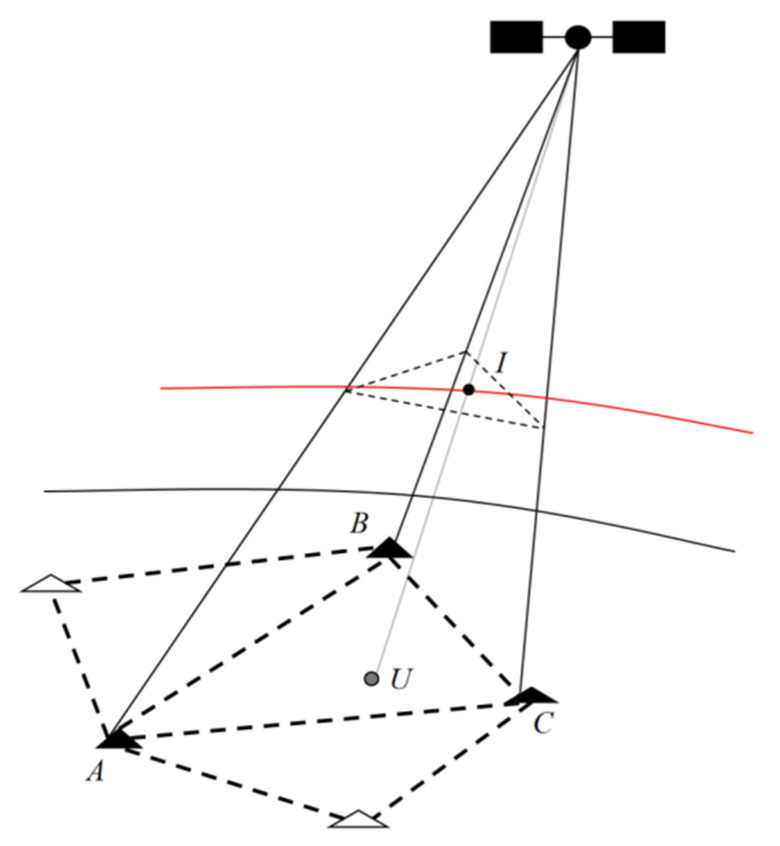
Schematic diagram of classification error interpolation.

Once the classified error components within the reference station network are determined, the corresponding classified correction values for the rover station can be obtained through appropriate interpolation algorithms. A schematic diagram of the classified regional error interpolation method is shown in Figure 7, where A, B, and C represent the reference stations, U denotes the rover station, and I indicates the ionospheric pierce point.

**Figure 8:**
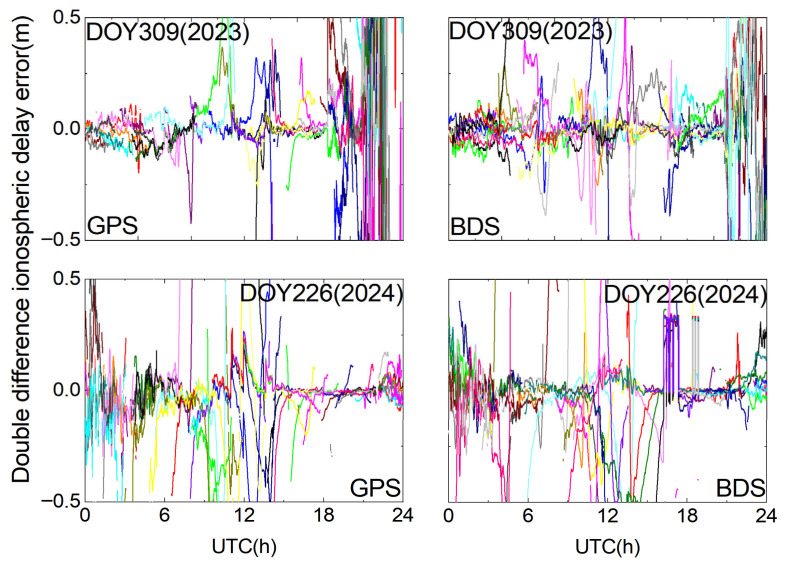
Double-differenced ionospheric delay on DOY 309 (2023) and DOY 226 (2024).

As shown in Figure 8, the double-differenced ionospheric delay variations in both the BDS and GPSs on DOY 309 (2023) are not completely stable. While the variations remain small and the trends are generally steady during most epochs, a significant increase in double-differenced ionospheric delay errors occurs around 12 h and between 21 and 24 h. This observation is closely related to disturbed plasma transport processes in the equatorial ionization anomaly (EIA) region and the occurrence of plasma bubble-induced scintillation during geomagnetic storms, which cause strong local ionospheric gradients and spatiotemporal decorrelation in GNSS observations. Such effects are particularly relevant for long-baseline RTK positioning from the user-side perspective, as they can severely degrade ambiguity fixing performance and increase positioning errors.

From Figure 1, it can be observed that the absolute value of Dst exhibits a sharp decrease (i.e., a strong enhancement in geomagnetic activity) between 10:00 and 12:00, followed by a persistently negative level from 12:00 to 24:00, with particularly intense disturbance during 21:00–24:00 on DOY 309 (2023). During this time, special attention must be paid to the impact of ionospheric delay errors on the ambiguity fixing performance of the reference stations. It is crucial to impose appropriate constraints on ionospheric parameters. The empirical power spectral density approach is evidently insufficient to capture the irregular intra-day ionospheric variations. Therefore, when fixing ambiguities at the reference stations, the temporal variability of ionospheric parameters should be taken into account. By adapting the power spectral density based on real-time estimation to reflect ionospheric changes, more reasonable constraints can be imposed, preventing excessive ionospheric fluctuations from hindering the integer ambiguity fixing at reference stations.

As shown in Figure 8, the double-differenced ionospheric delay variations in the BDS and GPSs on DOY 226 (2024) are less intense than those on DOY 309 (2023). However, noticeable variations are still observed between 0 and 4 h, and ionospheric disturbances affecting some satellites are also detected between 10 and 14 h, which are likely unrelated to geomagnetic storm activity. Although ionospheric disturbances can also occur during non-storm periods, they are typically regional, short-lived, and weak. In contrast, geomagnetic storms trigger large-scale, intense, and long-lasting disturbances that significantly degrade the spatial correlation of ionospheric delays across a CORS network. Therefore, this study focuses on geomagnetic storm-induced disturbances as the primary factor affecting long-baseline URTK performance, with non-storm periods considered only occasionally relevant for minor disturbances.

**Figure 9:**
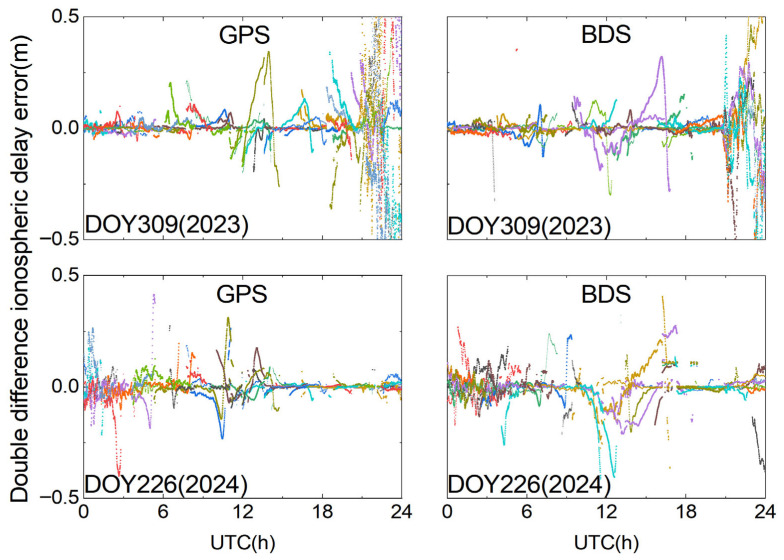
Double-differenced ionospheric delay accuracy at point U after interpolation.

To further verify the diurnal variation and accuracy of the ionospheric correction values received by the user under geomagnetic storm conditions, the double-differenced ionospheric delays at points A and U were first used as reference values. Thereafter, the ionospheric delays at point U were computed using interpolation of the double-differenced ionospheric delays from baselines AB and AC, and the difference between the reference and interpolated values was taken as the ionospheric interpolation accuracy at point U. The results show that during the geomagnetic storm, the differences between the interpolated and reference ionospheric delays at point U exhibited significant fluctuations and spikes. Although the degree of dispersion is somewhat reduced compared to Figure 9, it still indicates that under geomagnetic storm conditions, the accuracy of user-side double-differenced ionospheric corrections obtained through conventional interpolation methods is insufficient, necessitating additional estimation of atmospheric delay parameters on the user side.

**Figure 10:**
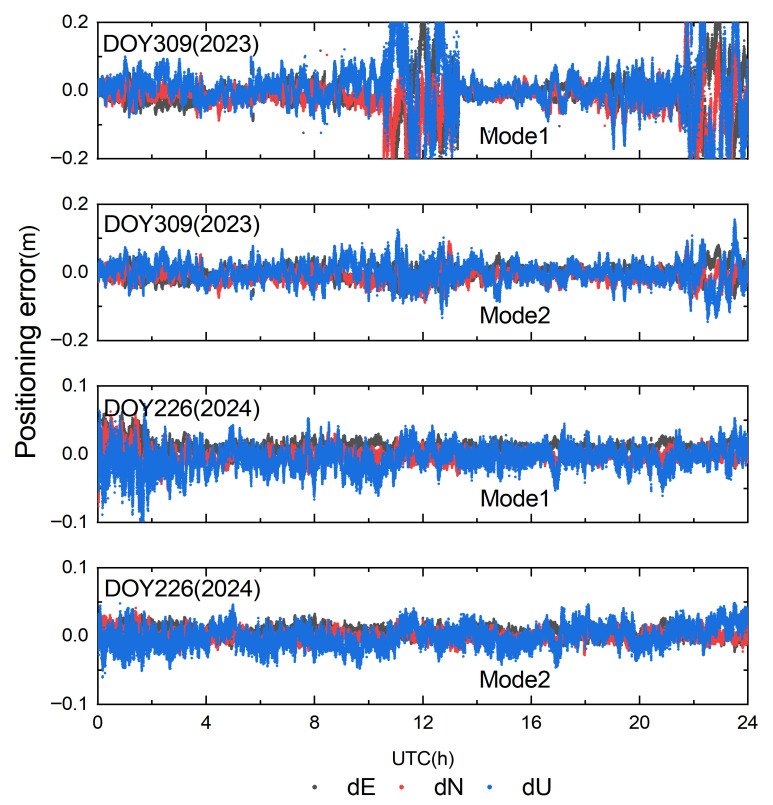
Positioning errors for the two positioning modes on DOY 309 (2023) and DOY 226 (2024).

In conventional network RTK, the user-side solution does not further process the ionospheric delay parameter; in our experiments, this conventional user-side solution is defined as Mode 1, with the user-side solution augmented with atmospheric parameter estimation defined as Mode 2. To compare the positioning performance of Modes 1 and 2 during geomagnetic storms and to obtain a quantitative understanding, the positioning results and the root-mean-square (RMS) statistics for the two modes on DOY 309 (2023) and DOY 226 (2024) are presented in Figure 10 and Table 2. From Figure 10, it is evident that during the geomagnetic storm on DOY 309 (2023), Mode 1 exhibits numerous outliers: the maximum positioning errors reach 28.2 cm in the E direction, 35.6 cm in the N direction, and 50.4 cm in the U direction. Mode 2 also shows some outliers during the storm period; however, for most epochs, the positioning errors remain within 10 cm relative to Mode 1. It is clear that Mode 1 struggles to meet high-accuracy positioning requirements during geomagnetic storms, whereas Mode 2 is scarcely affected; in non-storm periods, the positioning results of both modes are more stable. This finding is mainly the result of Mode 2 estimating residual atmospheric terms on the user side, greatly attenuating the impact of residual atmospheric errors—left after applying the regional undifferenced correction model—on ambiguity fixing. Likewise, the statistics presented in Table 2 clearly show that Mode 2 outperforms Mode 1 in positioning accuracy, with RMS improvements of 63.7%, 60.7%, and 64.4% in E, N, and U, respectively. During non-storm periods, the user-side positioning accuracy of Mode 1 can meet centimeter-level requirements, and its model complexity is lower than that of Mode 2. It should be noted that most current terminals still adopt Mode 1; thus, further implementation and verification on the terminal side are needed for future applications. Compared with Mode 1, Mode 2 improves the ambiguity fixing rate by 23.6% and 5.6% on DOY 309 (2023) and DOY 226 (2024), respectively.

Figure 10 shows the positioning errors on DOY 226 (2024). As seen in the figure, Mode 1 experiences pronounced fluctuations in the 0–3 h interval: the maximum positioning errors are 3.92 cm in E, 7.62 cm in N, and 11.0 cm in U, indicating that the user-side positioning is affected by the geomagnetic storm during this period. Over the same interval, Mode 2 is essentially unaffected, with RMS values in E, N, and U improving by 16.7%, 10.0%, and 11.1%, respectively, relative to Mode 1.

**Figure 11:**
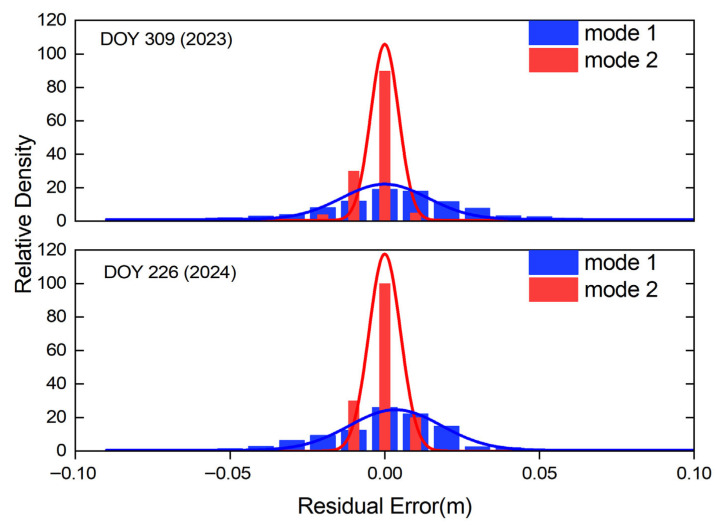
Normal distributions of residuals on DOY 309 (2023) and DOY 226 (2024).

To validate the positioning results, the post-fit positioning residuals of the two modes and their normal distributions are presented in Figure 11.

From Figure 11, clear differences can be seen in the relative density of the post-fit positioning residuals for the two modes on DOY 309 (2023) and DOY 226 (2024). Combined with the preceding analysis, this finding is mainly the result of Mode 1 having difficulty correctly fixing the user-side integer ambiguities during geomagnetic storms. Considering both the residual magnitudes and their normal distribution characteristics, most residuals in Mode 2 are within 1 cm, and the normal distribution of their relative density is markedly superior to that of Mode 1.

Comparing the results of this study with previous classical studies helps to better contextualize its contributions. Wielgosz et al. (2005) [12] analyzed the impact of strong ionospheric storms on long-baseline network RTK and found reduced correction accuracy and frequent instantaneous ambiguity fixing failures, which is consistent with the performance observed in Mode 1 in this study. Our results further demonstrate that incorporating user-side atmospheric parameter estimation (Mode 2) can effectively mitigate this issue, improving positioning accuracy by more than 60% under strong geomagnetic storm conditions and providing a concrete solution to this challenge.

Compared with the high-latitude study by Jacobsen and Schäfer (2012) [17], the results presented herein validate a different technical approach in low-latitude regions and provide more detailed quantitative improvement metrics. Furthermore, relative to studies on PPP and PPP-RTK, the URTK method examined in this study shows distinct advantages in terms of convergence speed and implementation complexity, offering an alternative and reliable solution for users with varying needs during highly dynamic space weather events [32,33].

## 4. Conclusions

In this study, we have investigated a long-baseline URTK method under geomagnetic storm conditions. Using CORS data from a low-latitude province in China and dual-constellation BDS + GPS processing, we have analyzed the server-side ambiguity fix rate and user-side positioning performance for DOY 309 (2023) and DOY 226 (2024). The main findings are as follows:

On the reference station side, employing an ambiguity resolution model with inter-epoch random walk constraints on additional atmospheric parameters enables accurate fixing of integer ambiguities under geomagnetic storms; for both days, most ambiguity fix rates exceed 90%.

On the user side, the positioning mode augmented with atmospheric parameter estimation achieves centimeter-level accuracy under geomagnetic storms and outperforms the conventional user-side solution in both strong and moderate storm conditions, with particularly pronounced improvements during strong storms.

In practical applications, the user-end software can be updated by embedding a user-side atmospheric delay estimation module, which can be manually activated when a geomagnetic storm occurs or when high-precision positioning becomes unreliable due to suspected ionospheric disturbances.

Despite the strengths of this study, several limitations remain. First, algorithm verification was conducted using data from only two geomagnetic storm events in a specific region, and its general applicability requires validation with more diverse datasets covering different regions and storm intensities. Second, the switching between the traditional mode and the user-side atmospheric parameter estimation mode has not yet been automated. In addition, the current model does not explicitly account for inter-station and inter-satellite ionospheric gradients, which can introduce significant interpolation errors in low-latitude ionospheric trough regions during geomagnetic storms.

To address these limitations, in future studies, we will focus on the following aspects:

(1) Collecting more diverse geomagnetic storm data for comprehensive generalization analysis;
(2) Developing intelligent mode-switching strategies based on real-time ionospheric activity indicators (e.g., ROTI and Dst indices) to enable an adaptive selection of user-side positioning modes;
(3) Incorporating ionospheric gradient modeling approaches such as that proposed by Liu et al. [34] to further enhance the stability and accuracy of network RTK under extreme space weather conditions.

## Figures and Tables

**Table 1:** Baseline information.

Baseline	Length (km)
A-B	92.7
B-C	97.5
C-A	119.5

**Table 2:** Statistics of RMS and ambiguity fixing rates for the two positioning modes.

Mode	E (m)	N (m)	U (m)	Ambiguity Fixing Rates (%)
Mode 1 DOY309(2023)	0.049	0.057	0.084	72.3
Mode 2 DOY309(2023)	0.018	0.022	0.030	94.6
Mode 1 DOY226(2024)	0.012	0.010	0.018	92.5
Mode 2 DOY226(2024)	0.010	0.009	0.016	97.9

## Data Availability

The data presented in this study are available from the corresponding author upon request. The data are not publicly available due to privacy restrictions. The authors gratefully acknowledge the Guangdong CORS network for providing the multi-GNSS data.
